# Classification of unknown primary tumors with a data-driven method based on a large microarray reference database

**DOI:** 10.1186/gm279

**Published:** 2011-10-17

**Authors:** Kalle A Ojala, Sami K Kilpinen, Olli P Kallioniemi

**Affiliations:** 1 Institute for Molecular Medicine Finland (FIMM), University of Helsinki, Tukholmankatu 8, 00140 Helsinki, Finland

## Abstract

We present a new method to analyze cancer of unknown primary origin (CUP) samples. Our method achieves good results with classification accuracy (88% leave-one-out cross validation for primary tumors from 56 categories, 78% for CUP samples), and can also be used to study CUP samples on a gene-by-gene basis. It is not tied to any *a priori *defined gene set as many previous methods, and is adaptable to emerging new information.

## Background

Cancer of unknown primary origin (CUP) is a classification given to a malignant neoplasm when a metastasis is discovered but the source of the primary tumor remains hidden. If counted together as a single clinical entity, CUP is one of the most common cancer types diagnosed in the world. Some 3 to 5% of all newly diagnosed cancers are CUPs, which qualifies this disease entity as one of the ten most common cancer types, with an incidence that is greater than that of, for example, leukemia or pancreatic cancers [1,2]. Even at autopsy, the location of the primary tumor remains a mystery in up to 70% of CUP cases [1,3]. CUPs present a significant challenge for physicians, since many of the current treatment regimes rely on knowledge of the type and origin of the primary tumor.

Several methods for identifying CUP samples based on their gene expression profiles have been developed. Talantov *et al. *[4] and Varadhachary *et al. *[5] presented an RT-PCR based method that measures the expression of ten signature genes. Ma *et al. *[6] proposed a similar method based on 92 genes, which resulted in an overall accuracy of 82% among 39 cancer types. Tothill *et al. *[7] presented a support vector machine-based method for classifying cancer types, and selected 79 genes for an RT-PCR test reaching a total accuracy of 89% but only among 13 cancer types. Rosenfeld *et al. *[8] applied a similar approach, but instead of measuring traditional gene expression, they looked at microRNA expression to classify CUP samples. For a majority of the samples, they achieved approximately 90% classification accuracy.

Since the development and adoption of gene expression microarrays, there has been interest in developing a microarray-based cancer classification, including a test to identify the origin of CUP cases. Microarrays provide a robust way to measure the expression of a large number of genes, and recently have been proven to be applicable in the clinical setting as well [9-12]. At least two custom microarrays are commercially available, CUPPrint by Agendia [13] and the Pathwork Diagnostics TOO test [14,15], and their validation data have been published [16,17]. Both tests utilize an *a priori *defined set of genes whose expression in the test sample is measured.

All the previous methods for identification of CUP tumors thus rely on a fixed set of training samples, sometimes with a narrow representation of histological types and anatomical sites, from which the informative genes have been determined. Thus, these methods cannot take into account the constantly accumulating scientific knowledge on gene expression across all types of cancers. Therefore, a more universal and adaptable method for microarray-based CUP prediction is desirable. If the identification of CUPs is performed algorithmically from genome-wide expression profiles, as opposed to from a defined gene list, the method is scalable, more flexible and open to improvement as reference data increase in both quality and quantity. Importantly, definitions of the histopathological and molecular subgroups of the reference tumors will dramatically influence the classifiers, possibly requiring major changes and improvements to existing disease classifications. For example, it may be important in the future to develop specific predictors for, for example, estrogen receptor-positive and -negative breast cancers, or the five major breast cancer subgroups, or for other very small subgroups, such as anaplastic lymphoma kinase-positive non-small cell lung cancers [18,19]. In other words, the scope of classifying the origin of CUPs will evolve rapidly as small subgroups of common cancers become better understood and it may become necessary to diagnose not just the origin of the primary tumor, but also the molecular subtype of the tumor.

Staub *et al*. [20] demonstrated that CUP prediction is possible using available microarray data from about 800 healthy samples and 600 cancer samples extracted from the Gene Expression Omnibus (GEO) [21] as a reference. They were able to construct a predictor using both cancer and healthy tissue samples. Their method is scalable, in that when new data become available, the genes used in the classifier can be re-evaluated. Although they achieved good accuracy (approximately 90%) in a leave-one-out cross-validation (LOOCV) test using primary tumors, the actual CUP prediction accuracy was only 60% in a small set of 20 test samples.

Here, we set out to create a CUP classifier that could easily be adapted to any reference data set. For this purpose, we analyzed test samples by aligning their microarray profiles against the annotated and normalized GeneSapiens microarray reference database and applied a slightly modified alignment of gene expression profiles (AGEP) method - weighted AGEP (wAGEP) - which we recently developed and described for classification of cell differentiation patterns [22]. The wAGEP method is described and validated in this paper.

## Materials and methods

### Study design

The aim of this study was to study CUP sample characterization using the previously published AGEP method [22]. The intent was to create a methodology suited not only to the classical problem of classifying the sample, but also one that would enable us to study CUP cases on a gene-by-gene basis. We wanted to be able to compare any gene's expression in the sample to reference data, and thus hopefully not only determine the tissue of origin, but also derive information relevant for treatment from the analysis.

### AGEP methodology

This study uses a modified version of the AGEP methodology. Briefly, AGEP calculates a tissue specificity score (ts-score) for each gene in a test sample for each predefined group (such as a tissue or cancer type) in the reference data. The ts-score measures, on a scale of -1 to 1, how well the gene's expression in the test sample classifies the sample as belonging to the group. A score of -1 indicates that, according to this gene, the sample is anything but a member of this group, while a score of 1 means a perfect fit to the group to the exclusion of all other groups. A score of 0 means the gene's expression is indeterminate when considering if the sample should belong to the group or not. A final similarity score between the test sample and each group in the reference data is then calculated taking the mean of all ts-scores for each group. The original AGEP algorithm can be found in [22].

### Gene uniqueness calculation

The weight for a gene in a particular cancer type was calculated as follows. First, density estimates for the gene for each cancer type in the reference data were constructed as demonstrated in [22]. We then examined the density estimate of the cancer type in question, and determined where it was higher than that of any other cancers. Within the range where the density estimate of the cancer in question was highest, we calculated the area between it and the next highest density estimate, regardless of what cancer type it represented (Additional file 1). Since all density estimates had their area normalized to 1, this procedure resulted in a number between 0 and 1, and represents the uniqueness of that gene's expression pattern in that cancer type when compared to all other cancer types.

### Gene weight application

Gene weights were applied as follows. When calculating the final similarity score between a test sample and a cancer type (mean of the ts-scores for each gene for that cancer type), each gene's ts-score was multiplied by the weight that gene had for the cancer type in question. The resulting ts-scores were then divided by the mean of all gene weights for that cancer type. This was done to normalize the different amounts of specific genes different cancer types possess. Finally, the similarity score between the test sample and the cancer type was calculated by taking a mean of the ts-scores. The workflow is depicted in Additional file 1.

### Reference database

Reference data, both expression values and annotation, were fetched from the GeneSapiens database [23]. The cancer data consisted of 5,577 samples that were grouped into 56 cancer types (Additional file 2). The healthy tissue reference data were the same as used in [22], consisting of 1,667 samples representing 44 tissue types.

### Test data

The test data were from GEO [21] study GSE12630. They were transformed to be compatible with the GeneSapiens database by using MAS5 and the equalization transformation as described previously [23,24]. Array-generation-based gene centering (AGC) was performed using the gene and array generation specific correction factors used to construct the GeneSapiens database.

### Data analysis

All data analysis was done with R [25].

### Accuracy versus best similarity score

**Figure 1:**
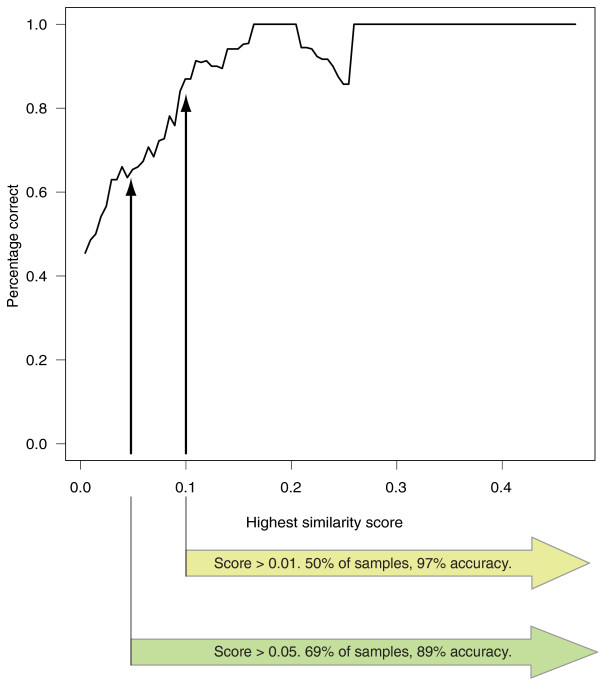
**A graph of the accuracy of the method as a function of the similarity score of the best hit**. The graph was formed by moving a sliding window of width 0.1 along the score axis, which ranges from -0.021 to 0.495, and calculating the achieved accuracy within that window. As can clearly be seen, the better the similarity, the higher the probability that the classification is correct.

The test samples were arranged according to the highest similarity score they had attained for any cancer, and whether this cancer was a correct classification was also recorded. From this, the fractions with the highest score above a certain threshold were trivial to calculate. A graph showing accuracy as a function of the highest similarity score was calculated using a sliding window. The width of the window was 0.1 (in similarity units) and it was moved in steps of length 0.005 over the ordered test sample population. The percentage of correct classifications within the window at each step was calculated (Figure 1).

### Heatmap and hierarchical clustering

Heatmaps were produced with the 'heatmap.2' function from the 'gplots' R library. Standard settings (Euclidean distance, complete linkage) were used for the hierarchical clustering of both genes and samples.

### AGEP and wAGEP functions

An R library that contains the original AGEP functionality and function for calculating and applying the gene weight (wAGEP portion) can be found at [26].

## Results

### AGEP method and its modification for CUP analysis

AGEP compares the expression value of a gene in a test sample to the distributions of expression levels of the same gene across all reference sample groups (for example, tissue or tumor types), and determines how well the expression level for the gene in the test sample fits with the corresponding distributions in the reference data. This analysis is then repeated for each gene. For a test sample, AGEP thereby provides a tissue match score (tm-score) for each gene for each reference tissue type, which quantifies how well that gene's expression corresponds to the levels in the reference tissue types. The AGEP method also evaluates how uniquely the tm-score categorizes the test sample among the tissues of the reference data. This is the tissue specificity score (ts-score). The output from an AGEP analysis are the tm- and ts-scores for each gene of the test sample in relation to each tissue type in the reference data. For a more in-depth description, please see Kilpinen *et al. *[22].

Tm- and ts-scores allow for comprehensive interpretation of the molecular nature of the query sample in relation to the entire reference dataset. For example, among healthy tissues, the tissue with the highest average ts-score for a test sample indicates the tissue of origin with high accuracy (93.6%) [22]. The original AGEP method considers each gene to be equally important when determining the similarity between a test sample and the reference data. In the case of cancer classifications, the search space is increased in both size and complexity. Cancers are composed of many more histological types and subtypes and most anatomically defined cancers are much more heterogeneous than their properly differentiated normal tissue counterparts. In order to further improve the tissue identification accuracy of the method, we applied an additional weight factor for each gene and for each cancer type in the reference data (resulting in the wAGEP method). This weight is based on the uniqueness of the gene's expression in each particular cancer type, and was added to strengthen the impact of highly predictive genes. The weight factor is derived from the density estimates for each gene, and is calculated from the area of the density estimate that is higher in the specific cancer type than in any other cancer type (Additional file 1), and is thus independent of the tm- and ts-scores. This weight ranges from zero to one, and is applied so that the tissue specificity score for each gene is multiplied by the appropriate weight before the final tissue similarity of the sample is considered. The entire workflow is depicted in Additional file 1, and further explained in the Materials and methods section.

The key advantage of the AGEP method is that it examines each gene of the test sample and each sample group (such as cancer types) in the reference database independently, and then compares the results across tissues to find the genes that best classify the test sample. This attribute is retained with the addition of the weight factor, and the weight only enhances the classifying potential of genes with cancer-specific expression profiles. Additional file 3 shows all the 17,730 genes used in this study, and their weight for each cancer type. As can be seen, most cancers have clusters of genes that are highly unique to them, and form the root of that cancer's histological identity. Therefore, as part of the effort to develop a reference set for CUP studies, we determined the most tumor-specific genes across all cancers.

The method used to determine gene weight gives, as expected, a high weight factor to genes already known to be highly expressed in certain cancers. For example, *KIT *in gastrointestinal stromal tumor (GIST; second highest weight in GIST, 0.95) and *KLK2 *and *KLK3 *in prostate cancer (the two highest weights in prostate adenocarcinoma, 0.97 and 0.95, respectively). Also, some new cancer-specific genes are found, such as *TMEM204*, which has the highest weight for GIST, 0.96; when looking at GeneSapiens [23] data, the gene's expression is shown to be extremely specific to GIST (Additional file 4). Overall, this set of cancer-specific genes could serve not only as a base for the bioinformatic analysis of CUP samples, but also as a starting point to develop tumor-specific biomarkers.

It is important to note that the classification is still based on all genes; some genes in each cancer type just have a bigger impact than others in determining the tissue specificity.

### Training data

We used the cancer samples from the GeneSapiens database as the reference data [23,24]. The data consist of 5,577 malignant tumor samples, whose gene expression microarray were all normalized to be directly comparable. The data represent 56 different cancer types, each class having an average of 100 samples per class, with a minimum of 6 (Additional file 2). Less than 1% of the samples were metastases; we thus refer to the reference data as primary cancer samples. These data were then used to construct cancer-specific gene density estimates for each gene in each of the 56 different cancer classes as described in Kilpinen *et al. *[22].

### LOOCV validation of the training data

To validate the integrity and applicability of the reference database for AGEP analysis, we performed a LOOCV analysis of the entire reference data. Thus, the tissue origins of all 5,577 individual malignant samples were analyzed by reconstructing cancer-specific gene density estimates without the sample in question. AGEP analysis revealed a total accuracy of 88.2% within the search space of 56 different *in vivo *cancer types when a match to similar cancer types was accepted or 79% if the exact match was required. Average sensitivity with the less strict criteria was 0.748 with a specificity of 0.999. Without the application of gene weights (general AGEP) the total accuracy of training data LOOCV was 78%, substantially less than with wAGEP (88%).

### Identification of the tissue of origin of CUP samples

Test data were from GEO [21] study GSE12630, which contains 187 metastases and poorly differentiated tumors (128 metastases and 59 poorly differentiated primary tumors).

**Figure 2:**
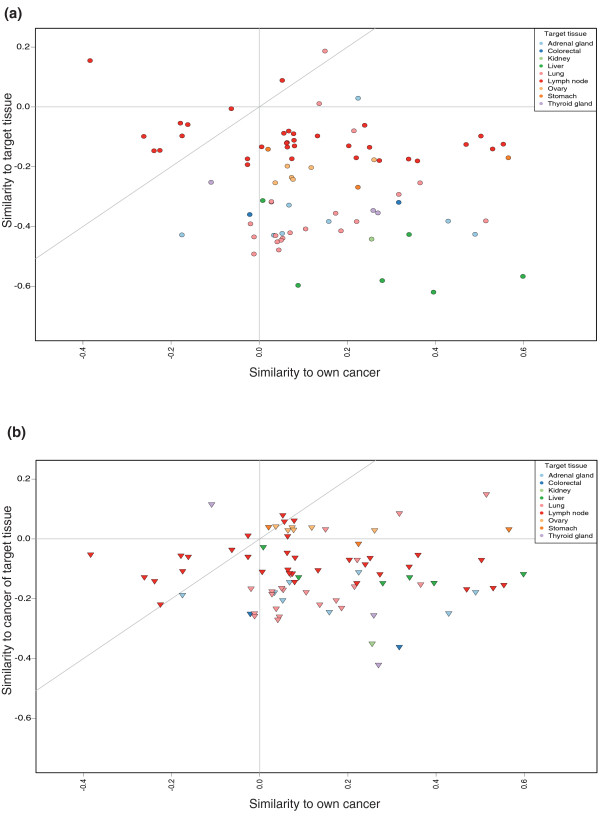
**Similarities for 83 metastatic test samples**. **(a) **A comparison of the test samples' similarities to the healthy tissue where the metastasis was found (y-axis) and the cancer of origin for the metastasis (x-axis). The spheres are colored according the site of the metastasis ('target tissue'). The gray diagonal line indicates a boundary, above which the similarity to the target tissue is greater than the similarity to the sample's original cancer. Only ten samples display this behavior, and all but one of these are lymph node metastases. **(b) **A comparison of the test samples' similarities to a representative cancer of the tissue where the metastasis was found (y-axis) and the cancer of origin for the metastasis (x-axis). The triangles are colored according to the site of the metastasis ('target tissue'). The gray diagonal line indicates a boundary above which the similarity to the cancer of the target tissue is greater than the similarity to the sample's original cancer. As can be seen, most samples fall below this line.

We originally compared the test samples against both the healthy tissue samples (1,667 samples in 44 healthy tissue types) and the 56 different cancer classes of the GeneSapiens database. The accuracy of prediction was 69% if we considered both appropriate healthy tissues and cancers as correct. Interestingly, we found that only 7% of the test samples had a healthy tissue group, as opposed to a primary cancer group, as their best match. This was the case for both test groups, the dedifferentiated primary tumors and metastases. We therefore conclude that the test samples, which imitate CUP problem solving, resemble cancers significantly more than their differentiated healthy tissue counterparts. As a consequence, subsequent analyses for this study were done by comparing the test samples only against the cancer reference data. Figure 2 illustrates the findings of the comparison of test samples against both healthy tissues and cancers.

Comparing the GSE12630 test set against reference tumors, we achieved 78.1% (78.1% for the metastases, and 78.0% for the primary tumor samples) total accuracy in identifying the tissue of origin. Classification was counted as accurate when (a) the cancer type with the highest similarity score was exactly the same as the test sample's annotation ('exact'); (b) when the cancer type with the highest similarity score was from the same organ, such as lung adenocarcinoma being identified as lung squamous cell carcinoma ('similar'); or (c) when the cancer type with the highest similarity score was from the anatomical site of the metastasis and the second highest cancer type was of category a or b above ('same site'). These results would all prompt a physician to consider the primary tumor in the correct anatomical site. Of the metastasis test samples, 64.8% were accurate according to definition a, 12.5% additional cases according to definition b and and additional 0.8% according to criteria c, resulting in a total accuracy of 78.1%. The percentages for the primary samples were 71.2%, 6.8% and not applicable (a sample from a primary tumor cannot fulfill this criterion), respectively, resulting in a total accuracy of 78.0%, with an average sensitivity of 72% and specificity of 99% across all samples (Table 1). The combined accuracies for each cancer type are shown in Table 1.

**Table 1:** Accuracies per cancer

Cancer	Total correct	Total samples	Percent correct	Sensitivity	Specificity
Bladder cancer	7	11	63.6%	64%	100%
Breast cancer	11	11	100%	100%	99%
Colorectal cancer	5	9	55.6%	54%	99%
Gastric cancer	10	15	66.7%	67%	98%
Liver cancer	6	8	75.0%	75%	100%
Lung cancer	14	15	93.3%	93%	95%
Lymphoma	23	25	92.0%	88%	99%
Melanoma	15	17	88.2%	88%	99%
Ovarian cancer	7	9	77.8%	78%	98%
Pancreatic cancer	4	13	30.8%	23%	99%
Prostate cancer	10	11	90.9%	91%	100%
Renal cancer	10	11	90.9%	91%	100%
Sarcoma	5	7	71.4%	71%	97%
Testicular cancer	13	16	81.3%	81%	100%
Thyroid cancer	6	9	66.7%	67%	100%
Total/average	146	187	78.1%	72%	99%

Numbers given for each cancer type are all samples correctly classified, all samples tested, the percentage of samples correctly classified as well as the sensitivity and specificity of the tissue of origin identification.

All but one cancer type showed at least 50% classification accuracy. The cancer that was particularly difficult to classify is pancreatic cancer, which is known to have a complex and heterogeneous genetic base [27]. Pancreatic cancer samples were often identified as esophageal cancers. Also, AGEP tends to confuse cancers originating from one part of the intestinal tract with cancers originating from another part of it. In fact, if we were to accept esophagus, gastric and colorectal as correct predictions for a cancer being of gastrointestinal origin, the total classification accuracy of gastric cancer would go from 66.7% to 93.3%, and that of colorectal cancer from 55.6% to 88.9%.

Interestingly, there is a strong correlation between the similarity score for the best match and the likelihood of it being correct. As can be seen from Figure 1, the higher the similarity score for the best hit among the reference data, the more likely it is to be correct. Thus, a low wAGEP similarity score means that the test sample does not resemble any of the cancers it is being compared to. It may be that the transcriptomic profile of a metastasis has deviated so much from its origin that it is more like an entirely new type of cancer. The apparent drop in accuracy around the value 0.2 seen in Figure 1 is due to a single gastric cancer metastasis sample being incorrectly classified as colorectal cancer. However, the annotation of the sample suggests that its real cancer type is at best an educated guess. If we were to ignore it, the resulting graph would rise steadily until it plateaued at around 0.15. Thus, we can assess the reliability of a wAGEP result simply by evaluating the similarity score of the best hit for that sample. If the highest similarity score for a cancer type is 0.1 or above (50% of test samples), the likelihood of the prediction being correct is 96.8%. If the score is 0.05 or higher (69% of test samples), the likelihood is still 89.1%. Conversely, if the score is lower than 0.05 (bottom 31%), the likelihood drops to 53.4%. Thus, it is advantageous not only to predict CUP tissue of origin, but also give an indication of how likely it is that the prediction is correct. The detailed results and original annotation for each sample can be seen in Additional file 5.

### Similarity to tissue of metastasis site

We also looked at whether the metastases would resemble the tissue where they were found. To do this, we returned to the comparison of the test samples versus the combined healthy and cancer data. Where possible, we determined the matching healthy target tissue to where the metastasis was detected ('target tissue') and a representative primary cancer of the same tissue ('cancer of target tissue') from the reference data. This was done for all metastasis samples. Of the 128 metastasis samples, 83 could be assigned to both a target tissue and a cancer of target tissue. We then studied whether the similarity of these test samples to either their target tissues or cancer of target tissue was dependent on any of the following: similarity to their original cancer, their cancer type, or the target tissue. In 62 of the 83 cases, the test sample's similarity to the cancer of target tissue was higher than its similarity to the target tissue. In all target tissues except lymph node the vast majority of the test samples resembled the cancer of target tissue more than the target tissue. In the case of the lymph node there was an about even split. In terms of the original cancer type, the results are similar. All other cancer types except thyroid carcinoma resemble their cancer of target tissue more often than the target tissue. For thyroid carcinoma, five out of the six samples resembled the target tissue more than the cancer of target tissue. However, four of these samples were lymph node metastases. The findings are not surprising, as any epithelial tumors metastasizing to lymph nodes will not start resembling lymphatic tissue derived cancers. The numbers for each target tissue and original cancer type can be seen in Tables 2 and 3.

**Table 2:** Numbers of metastasis samples that resemble the cancer of target tissue more than the target tissue, and vice versa, sorted per target tissue

Target tissue	Resembles target tissue more	Resembles cancer of target tissue more
Adrenal gland	1	7
Colorectal	1	1
Kidney	0	1
Liver	0	6
Lung	3	18
Lymph node	15	18
Ovary	0	6
Stomach	0	3
Thyroid gland	1	2
Total	21	62

**Table 3:** Numbers of metastasis samples that resemble the cancer of target tissue more than the target tissue, and vice versa, sorted per original cancer

Original cancer	Resembles target tissue more	Resembles cancer of target tissue more
B-cell lymphoma	1	5
Bladder cancer	2	7
Breast ductal cancer	1	6
Colorectal carcinoma	1	4
Gastric adenocarcinoma	0	9
Liver cancer	1	1
Lung adenocarcinoma	3	4
Lung, squamous cell carcinoma	0	2
Melanoma	0	9
Ovarian, endometrioid carcinoma	0	1
Ovarian, serous carcinoma	1	1
Pancreatic cancer	1	1
Prostate adenocarcinoma	1	2
Renal cancer	2	8
Testis, non-seminoma	1	1
Testis, seminoma	1	0
Thyroid cancer	5	1
Total	21	62

Figure 2 displays the similarities of the metastatic samples with their original cancer type, their target tissue and their cancer of target tissue. As can be seen, when the metastasis samples are compared against all-encompassing reference data, in over 80% of the cases (below the gray diagonal line) they still retain a higher similarity to their original cancer than to either their target tissue or their cancer of target tissue. A combined image for further study can be found in Additional file 6.

All these results reaffirmed our decision to analyze the test samples by comparing them to cancer only reference data.

### Cancer-specific genes

An advantage of the wAGEP method is that the results can be analyzed on a per gene basis. Thus, it is possible to identify the genes that would be good classifiers in the reference data (that is, genes that have a cancer-specific expression level) and explore whether those genes are useful in the identification of the metastasis samples.

**Figure 3:**
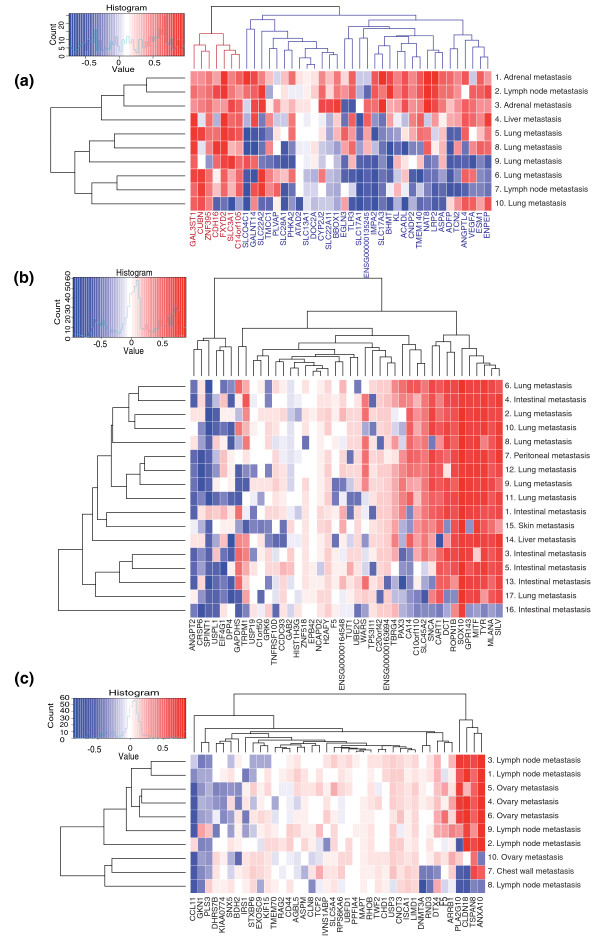
**Cancer-specific genes**. **(a) **Tissue specificity scores, unmodified by gene weight, for genes whose weight in renal cancer is greater than 0.25 (40 out of 58 present) are shown for 10 renal cancer metastasis samples. The genes can be divided into two groups, those that lose their renal cancer-specific expression (blue) and those that do not (red). The samples are named according to where the metastasis was located, and numbered according to their (relative to each other) similarity to renal cancer. Sample 10 was the only one whose closest similarity was not renal cancer, it instead being lung squamous cell carcinoma. Samples 1 to 3 are the closest to renal cancer, and retain for most of the genes renal cancer-specific expression levels. The other samples have lost renal cancer-specific expression among the genes with a blue background. **(b) **Similar analysis for the 17 metastatic melanoma samples, showing 42 (out of 63) genes. **(c) **Similar analysis for 10 metastatic gastric cancer samples, showing 40 (out of 53) genes.

We looked at the samples that were metastases of renal cancer from the test data, and specifically at genes having renal cancer-specific expression levels. There were 58 genes with gene weight >0.25 in renal cancer, and these were selected as the renal cancer-specific genes. Forty of these were present in all test samples. When their tissue specificity scores are plotted, a subset of genes are seen to loose their renal cancer-specific expression in the metastases (Figure 3a). The 40 genes can be divided into those that generally retain renal cancer-specific expression among all samples, and those that retain it only in the subset of samples (samples 1 to 3, indicated in blue in Figure 3a). Of note is that sample 10, a lung metastasis, did not have renal cancer as the closest match, instead identifying as lung squamous cell carcinoma.

The vast majority of the renal cancer-specific genes encode membrane bound proteins, such as the numerous solute carrier family (*SLC*) genes. The genes that retain their renal cancer-specific expression in all samples do not seem to differ strongly from the genes that do not. Of the genes that do not retain their renal cancer-specific expression in all samples a few are worth pointing out. One interesting gene is *CNDP2*, known to be overexpressed in renal cancer [28], but only in grade 1 and 2 cancers [29], with levels in grade 3 and 4 cancers being the same as those of normal tissues. When we examine the tm-scores obtained for this gene for each sample, a progression can be seen where those metastases that most closely resemble primary renal cancers have a high score for this gene, and as the samples diverge from the primary cancer, so does this gene's expression.

Also, the three angiogenesis-related genes, *ANGPTL4*, *VEGFA *and *ESM1*, seem to be expressed at their original levels in most samples and have altered expression in only a few samples. Finally, a group of three renal cancer-specific genes, *ATAD2*, *SLC13A1 *and *DOC2A*, seem to have lost their renal cancer-specific expression in all samples (all the samples are metastases), but the level of divergence from the renal cancer-specific expression seems to be stable, independent of the sample's overall similarity to renal cancer.

Similar analyses were done for melanoma (Figure 3b) and gastric cancer (Figure 3c). There were 17 metastasis samples of melanoma with 42 of 63 genes present, and 10 metastasis samples of gastric cancer with 40 of 53 genes present. In the melanoma case, we could see a group of genes that retained their melanoma-specific expression in some samples, and had lost it in others. However, the retention of melanoma-specific expression does not correlate well with either the sample's similarity to melanoma or the tissue where the metastasis was. Also, about half of the genes with melanoma-specific expression had altered expression in all the melanoma metastasis samples.

In the gastric cancer case we see a group of four genes, on the left side of the plot, which display different tm-scores between samples. In most samples the genes retain gastric cancer-specific expression, but in a few samples the genes' expression seems dramatically altered. As with the melanomas discussed above, most of the genes that have melanoma-specific expression seem to have lost that expression.

In both the melanoma and gastric cancer sample sets, one or two samples had completely lost their cancer-specific expression for all genes. These could be samples originally incorrectly annotated, or metastases that are dedifferentiated to the extent that they have no resemblance to their original cancer type.

## Discussion

Metastasis is an indicator of poor prognosis for any cancer patient, but the issue is even more difficult if the primary tumor is unknown and the diagnosis has to be made solely based on the discovery of metastases. This 'type' of cancer is known as a cancer of unknown primary (CUP) and represents a condition requiring specific clinical attention. The origin of the metastasis needs to be identified as primary treatment regimes for cancer are typically based on the anatomical origin and histological type of the primary tumor. Studies by several groups [4-7,20] have shown that finding the tissue of origin of metastatic samples is possible based on gene expression data. Some of these tests are already commercially available and have been clinically applied [13-15,17]. Most of the previously described approaches are based on a fixed set of genes measured with a custom designed array, multiplexed PCR or other molecular profiling assay. We sought to explore an approach where one can algorithmically solve the tissue of origin of the sample by comparing the whole genome expression profile of the sample to a large collection of reference data from the public domain, extracted from the GeneSapiens database [23]. This approach has the advantage of improving constantly as more data are acquired and as algorithms are optimized. This also allows more flexible customization of the molecular profiling to determine things such as where the metastasis originates from or whether the metastasis originates, for example, from esophagus or lung.

We show here that the wAGEP method is capable of identifying the tissue of origin of CUP samples with 89% accuracy when excluding the most uncertain 30% of the samples. If we, like some of the previously published studies have done [5], categorize any intestinal tract match as the correct classification for any tumor arising from that anatomical location, the accuracy increases substantially (by 26.7 to 33.3%). This is comparable to or better than what is achieved by most of the known methods, considering in particular the fact that we used one of the widest search spaces (56 different cancer types) compared to previous CUP studies [13-17]. The method can be improved in a data-driven way by adding more annotated reference data to the analysis. Thus, no specific gene selection or assay development is needed. Another key advantage of the wAGEP method is that it is able to determine how reliable the classification was. This would be helpful in a clinical setting when considering multiple treatment options for a patient in the context of, for example, contradicting diagnostic results from various tests.

Pancreatic cancer is quite common as a source of metastatic disease (between 25% and 12.5% of post-mortem identified CUP cases [3]), and it is the most difficult type of CUP tumor to identify using our method as well as all published methods [13-17]. Pancreatic cancer is often very poorly differentiated and progresses rapidly.

As the wAGEP method makes it possible to identify the tissue similarity as well as the genes behind the similarity, we were able to show which cancer-specific genes lose their cancer of origin-specific expression in metastatic samples (Figure 3). Even though each cancer is unique and metastatic progression and evolution are dependent on many variables, there were some systematic changes. To an extent, metastases maintain a similar transcriptomic program to that of the cancer of origin. This is reflected in the ability to identify the origin of metastases with reference data on primary tumors, but it is also visible at the level of individual genes (Figure 3). Further studies are also needed to uncover systematic changes in the transcriptomic program correlating with the site of metastasis. There are multiple studies indicating such changes, including *in vivo *mouse studies [30]. However, the currently available datasets of *in vivo *metastatic samples are still too few in number and size to allow systematic studies of this subject

The ability to directly interpret expression profiles of CUP tumors using a constantly increasing body of scientific data and knowledge allows for a faster and more economical way of providing more accurate diagnostics for CUP patients. This is essential as having metastatic carcinoma of unknown origin is a difficult situation for cancer patients; the average survival of these patients is only a few months [1]. Application of the proposed method needs a microarray-based expression profile from the metastasis, but several large hospitals and institutions around the world have already developed infrastructure for genomic and molecular profiling of tumors. Also, microarray technology in general is mature and, for example, Affymetrix genechips have been found to be a clinically applicable and robust platform [9-11,31-33]. Our method is Affymetrix-based, but could equally well be adapted for other platforms.

Full genome microarray analyses of CUP patients, like of any cancer patient, will also provide more information than just the tissue of origin. As the poor survival statistics of metastatic cancer patients show, CUP patients would need more than just the identification of the origin of metastasis. The wAGEP approach will provide data on the expression of all genes in the metastatic tumor, including information on potentially important subgroups or the expression of therapeutic targets that could be simultaneously assessed. Tailored medication based on these observations might prove to be a more useful approach than the traditional approach of anatomy- and histology-based treatment regimes.

## Conclusions

The wAGEP method proved to be good for classifying CUP samples. More than that, however, it showed that it was capable of finding and analyzing differences between the metastasis samples and their primary cancer types, thus providing interesting information that could have clinical significance. It is also not tied to any predefined gene list, or indeed anything predefined. It is fully scalable and able to adapt to new emerging scientific data.

## Abbreviations

AGEP: alignment of gene expression profiles; CUP: cancer of unknown primary origin; GEO: Gene Expression Omnibus; GIST: gastrointestinal stromal tumor; LOOCV: leave-one-out cross-validation; tm-score: tissue match score; ts-score: tissue specificity score; wAGEP: weighted AGEP.

## Competing interests

KAO and SKK are inventors on a patent application regarding the original AGEP method. SKK and OPK are shareholders in Medisapiens Ltd, which develops microarray data analysis technologies.

## Authors' contributions

KAO designed the study, performed the majority of the work and wrote the manuscript. SKK did the LOOCV validation part, provided ideas for the project and participated in the writing of the manuscript. OPK supervised the project. All authors have read and approved the manuscript for publication.

## Supplementary Material

Additional file 1 **An illustration of the method used to calculate similarities between a test sample and the reference data**. **(a) **The weight of a gene for each cancer type in the reference data is calculated by taking the area where the gene's density estimate is higher than that of any other cancer. Since the area under each density estimate is 1, the resulting weights are numbers between 0 and 1. The weights are unique for each cancer, and represent the ability of the gene's expression to distinguish that cancer from all others. **(b) **A schematic of the process for calculating the similarity between a test sample and the reference data. The AGEP procedure is modified by having the gene weights calculated from the density estimates and applied to the ts-scores of the normal AGEP result. Then, as per normal AGEP procedure, the tissue similarity for the test sample and each cancer in the reference data is calculated by averaging the now weighted ts-scores for that cancer. Click here for file

Additional file 2 **A summary of the reference data, the name of each cancer type and the number of samples it has**. Click here for file

Additional file 3 **Heatmap of all genes and all cancers used in the analyses. Genes are colored according to their weight**. Click here for file

Additional file 4 **GeneSapiens boxplot of the *TMEM204 *gene**. Click here for file

Additional file 5 **Results for each individual test sample**. Each sample is annotated as accurately as possible, and the five highest similarity scores and their corresponding cancer types are shown. Click here for file

Additional file 6 **A combination of the two images from Figure **2. Similarities of 83 metastatic test samples. Displayed are the similarities to the samples' own cancer, the tissue where the metastasis was found and a representative cancer of that tissue. The x-axis indicates the similarity of the sample to its cancer of origin. On the y-axis, a sphere indicates similarity to the healthy tissue where the metastasis was found. A triangle indicates similarity to a representative cancer of that tissue. The vertical lines are simply connectors for ease of visualization, indicating which sphere and triangle represent the same sample. If the line is solid, the test sample has a higher similarity to the cancer of the target tissue than the target healthy tissue, and *vice versa *if the line is dashed. The icons are colored based on the tissue where the metastasis was. Click here for file
