# Supplementary figures and images for: Classification of unknown primary tumors with a data-driven method based on a large microarray reference database
Source: Genome Med. 2011 Oct 17;3(9):63. doi: 10.1186/gm279 (PMC3239238; doi:10.1186/gm279)

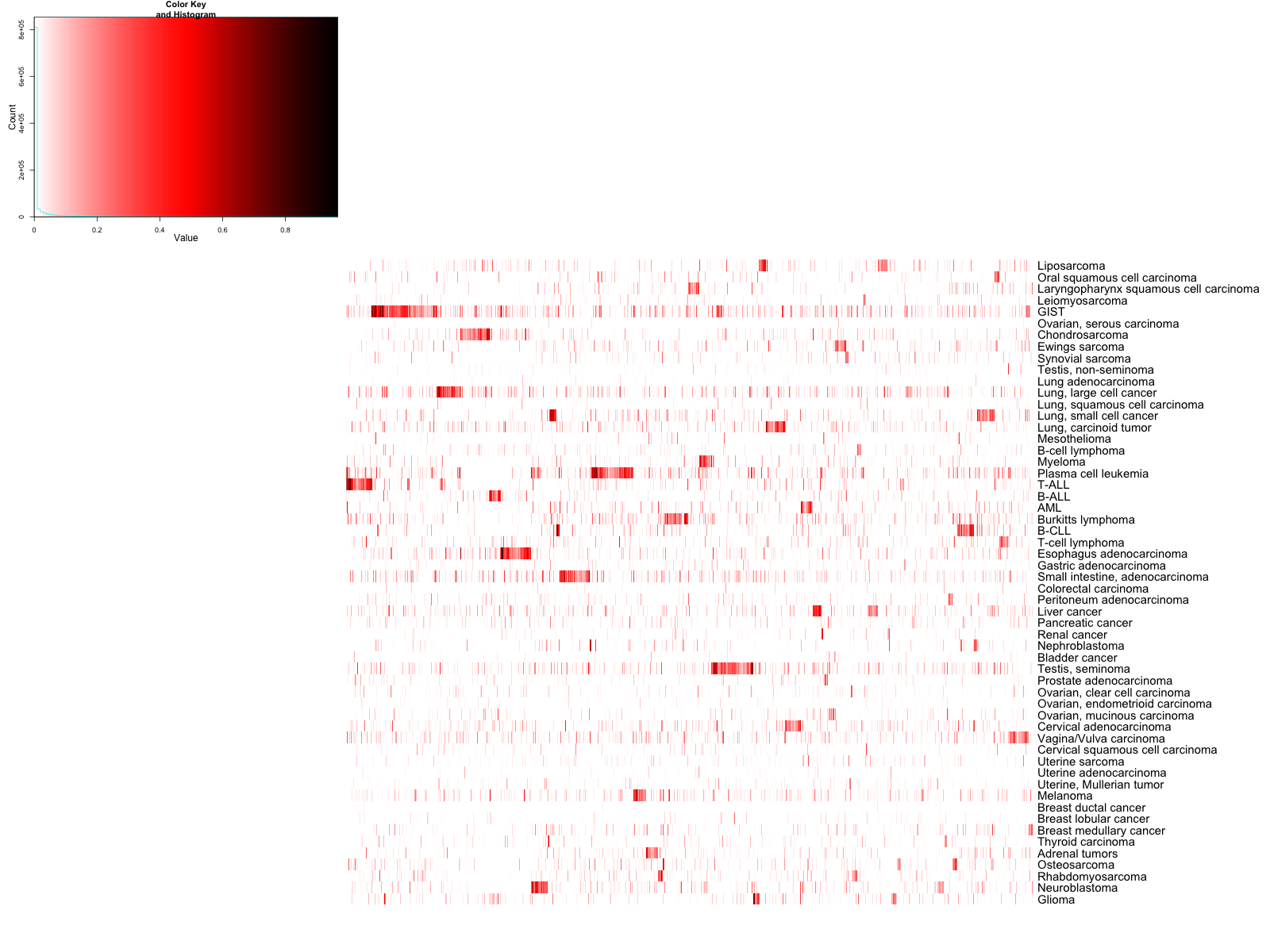

Supplement: Additional file 3 — Heatmap of all genes and all cancers used in the analyses. Genes are colored according to their weight. [file gm279-S3.PNG]
